# Computational Support to Explore Ternary Solid Dispersions of Challenging Drugs Using Coformer and Hydroxypropyl Cellulose

**DOI:** 10.1021/acs.molpharmaceut.4c00592

**Published:** 2024-10-10

**Authors:** Andreas Niederquell, Susanne Herzig, Monica Schönenberger, Edmont Stoyanov, Martin Kuentz

**Affiliations:** † Institute for Pharma Technology, University of Applied Sciences and Arts Northwestern Switzerland, School of Life Sciences FHNW, Hofackerstr. 30, 4132 Muttenz, Switzerland; ‡ Nano Imaging Lab, Swiss Nanoscience Institute, University of Basel, Klingelbergstrasse 50, 4056 Basel, Switzerland; § Nisso Chemical Europe, Berliner Allee 42, 40212 Düsseldorf, Germany

**Keywords:** solid dispersion, hot melt extrusion, molecular interactions, hydroxypropyl cellulose, coformer

## Abstract

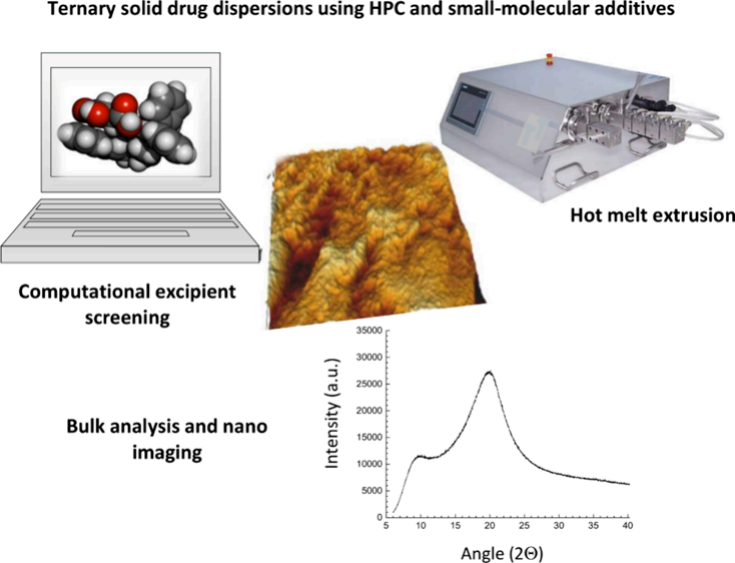

A majority of drugs marketed in amorphous formulations have a good glass-forming ability, while compounds less stable in the amorphous state still pose a formulation challenge. This work explores ternary solid dispersions of two model drugs with a polymer (i.e., hydroxypropyl cellulose) and a coformer as stabilizing excipients. The aim was to introduce a computational approach by preselecting additives using solubility parameter intervals (i.e., overlap range of solubility parameter, ORSP) followed by more advanced COSMO-RS theory modeling. Thus, a mapping of calculated mixing enthalpy and melting points is proposed for *in silico* evaluation prior to hot melt extrusion. Following experimental testing of process feasibility, the selected formulations were tested for their physical stability using conventional bulk analytics and by confocal laser scanning and atomic force microscopy imaging. In line with the *in silico* screening, dl-malic and l-tartaric acid (20%, w/w) in HPC formulations showed no signs of early drug crystallization after 3 months. However, l-tartaric acid formulations displayed few crystals on the surface, which was likely a humidity-induced surface phenomenon. Although more research is needed, the conclusion is that the proposed computational small-scale extrusion approach of ternary solid dispersion has great potential in the formulation development of challenging drugs.

## 1 Introduction

Novel drug candidates are often poorly water-soluble, entailing problems with erratic and variable drug absorption as well as possible food effects, which generally require so-called bioenabling formulations.^1−3^ Commonly used are solid dispersions, which are mostly about polymer-based amorphous dispersions, and excellent reviews have been written on this topic.^4,5,6^ The noncrystalline form of the active pharmaceutical ingredient (API) can generate supersaturation depending on the formulation matrix, which typically promotes drug absorption in the gastro-intestinal tract.^7,8^ Apart from this biopharmaceutical formulation performance, a solid dispersion must of course be physically stable for the entire shelf life of the drug product, and compounds pose different challenges in achieving this objective. A drug classification has previously been introduced that differentiates compounds based on the glass-forming ability (GFA) of their undercooled melt.^9^ While some compounds are practically nonglass-forming (class I), at the other end of the GFA spectrum are so-called good glass formers (class III). There is a further class between these extremes for compounds that form glass from the melt but display recrystallization in the second heating of the standard differential scanning calorimetry method.^10,11^ This compound GFA appears to be an intrinsic drug property, relevant also for amorphization processes other than cooling from a melt^12^ and for the storage stability of both the drug alone and when formulated with polymers as an amorphous solid dispersion.^13,14^

The vast majority of amorphous solid dispersions (ASDs) on the market are good glass formers (i.e., class III).^15^ It is therefore common that for GFA I and II compounds, other bioenabling formulations than ASDs are considered.^16,17^ Class II compounds could still be realistically stabilized in amorphous form, but this strategy is risky and a single matrix-forming excipient may not be sufficient to obtain a stable drug product. There are examples of using two polymers instead of a single matrix former to achieve increased ASD stability.^18,19^ Apart from stability considerations, such ternary ASDs can show further biopharmaceutical advantages.^20,21^ Ternary ASDs may not only comprise an additional polymer but, instead, other excipients can be added, so a classification according to composition has been given previously.^22^ Curiously, the addition of such a small-molecular excipient with the primary aim of drug stabilization is a more recent approach.^23^ This is similar to the neighboring field of so-called coamorphous systems, but the latter formulations are different in terms of composition and manufacturing process because there is no polymeric matrix as it is otherwise present in modern ternary ASDs.^24,25^ However, due to the biopharmaceutical advantages of polymers as drug precipitation inhibitors,^26,27^ recent work on coamorphous systems advocated a small amount of polymer (5–10%, w/w) in the mixtures, but the process was still ball-milling because there was not enough polymer for hot melt extrusion that is actually preferred for lean manufacturing.^28−30^ Therefore, the use of ternary ASDs with a small-molecular additive that exhibits strong drug interactions, i.e., a coformer, is an interesting concept. This type of ternary ASD could be especially interesting regarding GFA II drugs. However, such a tailored formulation strategy with various combination options would require either much additional experimentation compared to ordinary ASD development or digital tools should be harnessed to guide formulators. The present paper therefore addressed this computational screening need as another main objective.

Various digital methods have been tried for the development of ASDs and an overview of methods ranging from solubility parameters, Flory–Huggins theory, and molecular simulations to machine learning can be inferred from a recent review.^31^ Interesting is especially the conductor-like screening model for real solvents (COSMO-RS) as it combines quantum-chemical calculations with statistical thermodynamics.^32,33^ The model has been used for a modern estimation of solubility parameters, solubility, and solvate and cocrystal prediction.^34−36^ The model has been further introduced to the field of coamorphous systems, and most recently, entire drug-polymer phase diagrams were predicted by COSMO-RS.^37−39^ However, such modeling requires adaptations for solid dispersions of multiple components in an industrial screening context and regarding the specific manufacturing requirements of melt extrusion.^40^

This paper introduces the concept of an overlap range of solubility parameter (ORSP) to preselect coformers that are then further evaluated calculating (COSMO-RS) excess enthalpy, *H*_ex_, of the drug and coformer. These latter values are then mapped against the melting points of the coformers by considering the process temperature interval of the preferred polymer. Hydroxypropyl cellulose was selected here as a model together with the challenging GFA II drugs of bifonazole (basic p*K*_a_ = 6.28; log *P* = 5.23) and cinnarizine (basic p*K*_a_ = 7.75; log *P* = 5.88).^11,41^ Following the computational preformulation, selected ternary solid dispersion candidates were studied for their stability by differential scanning calorimetry (DSC) and powder X-ray diffraction analysis (XRPD). Moreover, confocal laser scanning and atomic force microscopy were employed as further analytics because of their known sensitivity to early on detect possible recrystallization during storage.^42,43^

## 2 Materials and Methods

### 2.1 Materials

The pharmaceutical active ingredients bifonazole and cinnarizine, as well as l-ornithine hydrochloride, were obtained from Biosinth Ltd. (Compton, Great Britain). Citric acid (99%), dl-malic acid, and l-tartaric acid were purchased from Sigma-Aldrich Chemie Ltd. (Steinheim, Germany). The polymers HPC L grade (∼140 kDa), grade SL (∼100 kDa), and grade SSL (∼40 kDa) were kindly donated by Nippon Soda Co., Ltd. (Tokyo, Japan). All materials were used as received.

### 2.2 Methods

#### 2.2.1 Quantum-Chemical and Thermodynamic Modeling to Determine Mixing Enthalpies

The theory of COSMO-RS was introduced by Prof. A. Klamt, and it is based on a solute embedded in a (conducting) continuum.^32,33^ A three-dimensional molecular geometry with respective surface charges is determined by means of quantum chemical calculations. Accordingly, the electron density is converged to its energetically optimal state in the assumed conductor to obtain surface screening charge densities σ that are used for further calculations.^33^

COSMO-RS considers a solvent *S* as an ensemble of pairwise-interacting surface segments. The interaction energies of the surface pairs are defined in terms of the screening charge densities σ and σ′ of the respective surface segments. The so-called σ profile is used for each component and is essentially a histogram of the respective screening charge densities *p*^*x*^(σ). Subsequent thermodynamics calculations require equations for the relevant electrostatic, hydrogen bonding, and van der Waals interactions, whereby a pseudochemical potential  of a compound *X*_*i*_ in the system *S* can be calculated by integration of μ_*s*_(σ) over the surface of the compound:^44,45,41^
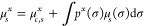
1

There are of course differences in the size and shape of the molecules in the system, so an area- and volume-dependent combinatorial term  is further considered in the equation above; the required molecular surface areas and volumes are taken from the COSMO cavities.

Because quantum chemical calculations based on density functional theory (DFT) are computationally intensive, the present work utilized a fragment-based approach to estimate sigma surfaces based on a large database of DFT results at a valence triple-ζ polarization (TZVP) level of theory.^46^ Accordingly, the program COSMOquick (v.2020, Biovia, Dassault Systèmes) was used for all of the COSMO-based calculations in the present work. In line with the outlined workflow of thermodynamic property calculations, the mixing enthalpy was finally determined by the following equation:
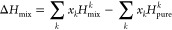
2where *x*_*k*_ stands for the mole fraction of a component *k*,  is the enthalpy of compound *k* in the mixture, and  represents the enthalpy of the pure component *k*. Thus, Δ*H*_mix_ denotes the enthalpy change during mixing of components as compared to their pure liquid state and, in the case of solid components, this can be viewed as an intermediate state of supercooled liquid components.^35,21^ A 1:1 molar ratio of the drug and coformer and 25 °C were selected for calculating Δ*H*_mix_ that is used in the following interchangeably with the term excess enthalpy, *H*_ex_.^35^ Information on typical values for mixtures of drugs and excipients can be found in the literature.^38,47^

#### 2.2.2 Calculation of Solubility Parameters

The concept of solubility parameters has been used extensively in pharmaceutical sciences, and the different methods as well as relevant applications have been reviewed previously.^48^ COSMO-RS also provides a framework to estimate solubility parameters, either via an empirical relationship using COSMO moments or via solubility value calculations under different conditions.^34^ The latter computational method is close to a common experimental approach except that solubility values are calculated *in silico*. Accordingly, a virtual solubility screening using 29 reference solvents provides an initial estimate of the partial solubility parameters to compute the activity coefficient ln(γ) as follows:^34^
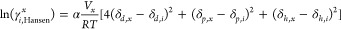
3where *x* refers to the solute (i.e., active compound), *i* stands for a reference solvent, α is the universal (Hansen) parameter, and *V*_*x*_ is the molar volume of the solute. Subsequently, activity coefficients are computed via COSMO-RS and partial solubility parameters in eq 3 are varied to minimize squared differences according to eq 4:^34^
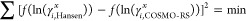
4

The function *f*(ln(γ)) is sigmoidal, and it was introduced to discriminate between good (when the function takes values close to unity) and bad solvents (for which values are close to zero); more information on the accuracy of the method to characterize drugs can be inferred from the literature.^34^

#### 2.2.3 Hot Melt Extrusion

The experimental extrusion was then conducted on a small-scale ZE9 ECO twin-screw extruder by ThreeTec Ltd. (Birren, Switzerland) with corotating screws of 9 mm in diameter and 180 mm in length with the standard configuration consisting of a conveying zone, a melting and mixing zone, and a discharging zone. The screws were encased in the extrusion barrel, which contained three individual heating zones. The extrusion temperature was set to 150 °C and 15–20 °C higher for the formulations with the coformer tartaric acid. After stable temperatures were reached, the screw speed was set to 50 rpm, all ingredients were premixed with a pestle and mortar, and the blend (minimum of 5 g) was then added manually to the extruder in small portions. The extrudates were cooled, sealed in aluminum bags, and stored at 25 °C, 60% relative humidity, and RH.

#### 2.2.4 Differential Scanning Calorimetry

Extrudate samples were assessed by differential scanning calorimetry on a DSC 3 instrument (Mettler Toledo, Greifensee, Switzerland). A 5 to 9 mg sample was placed in a 40 μL aluminum pan with a pierced lid. A heating/cooling rate of 10 °C/min from 10 to 220 °C was applied under nitrogen purging at 200 mL/min. A whole measurement included two cycles of heating and cooling within the specified temperature range and rate. The thermograms were analyzed with STARe evaluation software version 16 (Mettler Toledo, Greifensee, Switzerland).

#### 2.2.5 X-ray Powder Diffraction Analysis

To obtain a powdered sample, extrudates were first cut into small pieces and ground with a Freezer/Mill cryogenic grinder from Spex SamplePrep LLC (Metuchen, USA). After filling the device with liquid nitrogen from Carbagas Plc (Muttenz, Switzerland), the cylindrical sample holder, including approximately 2 g of extrudate pieces and a metal rod for milling, was inserted into the grinder. The precooling option was set to 10 min with a milling rate of 10 counts per second for 2 cycles of 2 min each. The resulting powder was immediately purged with argon gas from Carbagas AG (Muttenz, Switzerland), packed and sealed in airtight aluminum bags, and stored in a fridge at 4 °C.

The cryomilled extrudate samples were analyzed by X-ray powder diffraction (XRPD) analysis and compared to the untreated neat drug. The analytical X-ray diffraction patterns were obtained using an X-ray diffractometer (D2 Phaser) from Bruker AXS Ltd. (Karlsruhe, Germany) equipped with a Cu KFL tube as a radiation source (30 kV, 10 mA, and a radiation wavelength of 1.542 Å) and a 1D Lynxeye detector. Each sample was automatically rotated on a sample holder at 15 rpm, and the angular scanning range for each sample was from 6° (2θ) to 42° (2θ) with a 0.016° step size (2θ) at 2.0 s per step.

#### 2.2.6 3D Laser Scanning Confocal Microscopy

Laser scanning micrographs of the sample surfaces were collected by means of a 3D laser scanning confocal microscope (LSM) VK-X1100 (Keyence, Osaka, Japan) using a violet laser (404 nm) and a 150× objective lens (Nikon Plan CF Apo, 150×/0.95, WD 0.2 mm). While the surface was scanned at high speed in *X* and *Y*, allowing image capture with high lateral resolution, the lens moved in the *Z* direction and recorded the intensity of the reflected light. A peak search technology was used as a proprietary algorithm to calculate an appropriate *I*–*Z* curve from the obtained reflection intensity data, including the reflection intensity between steps, and determined the height position with the maximum intensity over the entire range of the *Z* direction. The intensity of the laser light that passed through a pinhole was determined by a very sensitive 16-bit photomultiplier. Since the pinhole blocks most of the returning light except that from the focal point, confocal LSM delivers much sharper images than conventional microscopy techniques. The lateral resolution was measured at 130 nm, whereby the distances between black and white lines written on a flat mirror surface were resolved. The general scan area measured was 70 × 90 μm with a pixel size of 768 × 1024. Finally, a true color image from the integrated second light source was overlaid.

#### 2.2.7 Atomic Force Microscopy

Atomic force microscopy (AFM) images of extrudates were acquired under ambient conditions in dynamic AC mode using a JPK NanoWizard 4 AFM instrument (Bruker Nano GmbH, Berlin, Germany). Height and phase images were collected simultaneously using a standard silicon tapping mode AFM cantilever (type 160AC-NA, OPUS by MikroMasch, NanoAndMore GmbH, Wetzlar, Germany) with a nominal length of 160 μm, a nominal resonance frequency of 300 kHz, a nominal force constant of 26 N/m, and an aluminum reflective coating. Regarding AFM resolution, this is generally the minimum motion that can be controlled and measured in the *x*-, *y*-, and *z*-axes, but also the geometry of the probe is decisive for lateral resolution. The used AFM instrument typically achieves a lateral resolution of a few nanometers and a vertical resolution in the range of a few hundreds of picometers under the given conditions. A representative scan area in this study was 10 × 10 μm at 512 × 512 pixels.

## 3 Results and Discussion

### 3.1 Computational Excipient Screening

This study aimed to formulate unstable glass-forming (i.e., GFA II) model drugs by means of ternary solid dispersions while introducing a computational screening method to guide formulators. Polymer-based solid dispersions are among the preferred formulation principles if a compound has a good glass-forming ability.^17^ Other GFA class I or II compounds rarely make it to the market as an ASD because of the low probability of stabilizing the drug in amorphous form during the entire shelf life.

There are different drivers of drug crystallization, starting from thermodynamic, kinetic, and environmental factors, as reviewed elsewhere.^49^ This mechanistic complexity certainly poses a hurdle regarding absolute predictions of physical stability, but for screening purposes, some approaches have still been found useful. One such classical approach is the use of solubility parameters to predict the likely miscibility of the components.^48^ Thus, the difference of the total (i.e., Hildebrand) solubility parameter of the drug and excipient should not be greater than 10 MPa^0.5^, as this is associated with immiscibility.^50,51^ In an interval of ±7 MPa^0.5^, there is likely full miscibility of the components,^51^ leaving the differences between 7 and 10 MPa^0.5^ for typically miscibility depending on the specific ratios (i.e., partial miscibility). This work suggests the use of the total solubility parameter for a preselection of coformer candidates to reduce the computational burden for a next step based on the more advanced COSMO-RS model.

For such a first screening, the present work introduces the concept of the overlap range of solubility parameter (ORSP). Although solubility parameters have been considered for multicomponent solid dispersions,^52^ the concept of ORSP as preselection for a subsequent more sophisticated excipient selection is new to the best of our knowledge.

**Figure 1:**
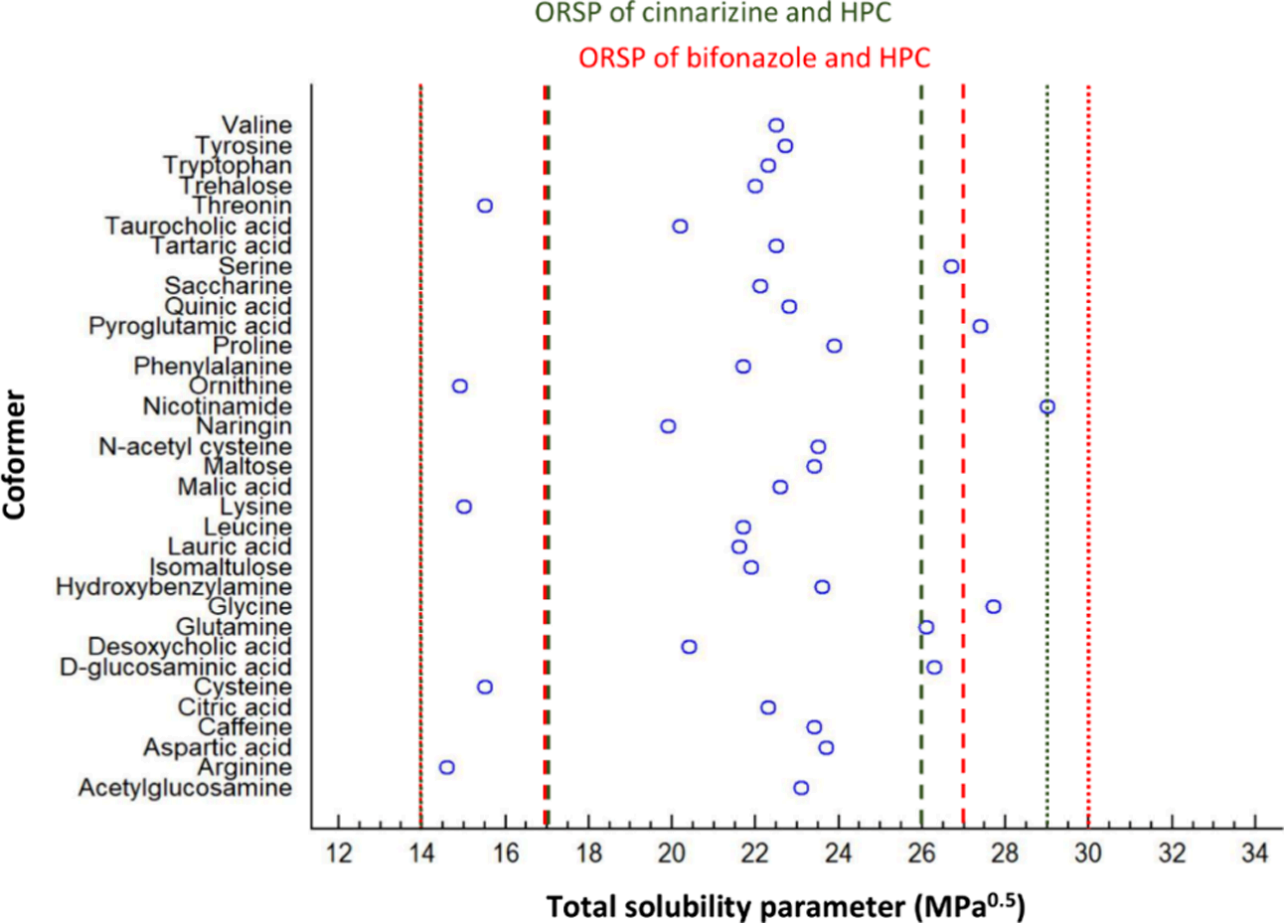
Calculated solubility parameters (MPa^0.5^) for a range of coformers that were selected based on the overlap range of solubility parameter (ORSP) of the model drugs and hydroxypropyl cellulose (HPC). Limits of the ORSP are given both for a broader (dotted lines) or more restrictive interval (dashed lines) for cinnarizine (in green) and bifonazole (in red).

**Figure 2:**
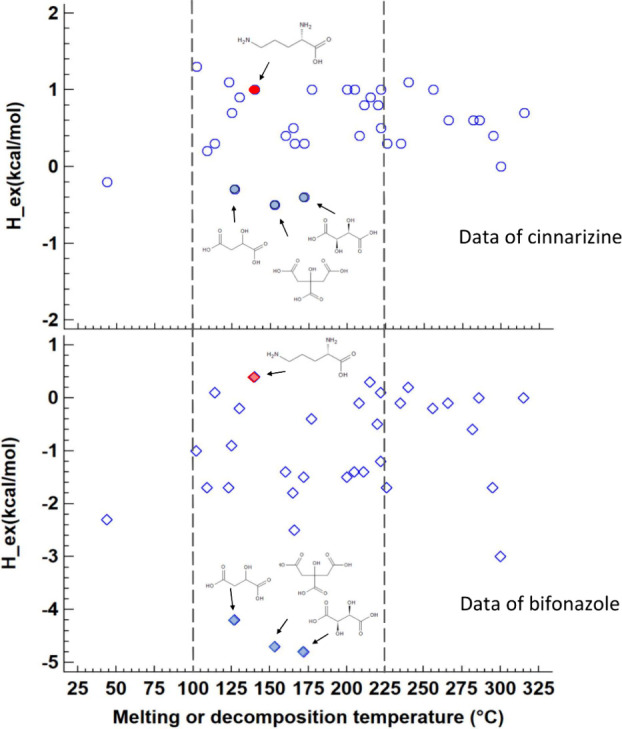
Predicted excess enthalpy, *H*_ex_, of the various coformers with model drugs (equimolar mixtures) plotted versus the melting or decomposition temperature of the additives. Most promising coformers were found for a group of three acids in order of increasing melting points: malic, citric, and tartaric acids. These preferred coformers (filled symbols in blue) were further investigated together with the negative control additive ornithine (filled symbols in red). The dashed lines indicate a typical operating window for hot melt extrusion with HPC.

A recently published computational method was used to predict the total solubility parameter:^34^ 19 MPa^0.5^ was found in the case of cinnarizine and 20 MPa^0.5^ for bifonazole. The current work focused on HPC as a polymer based on recent biopharmaceutical interest in the field of hot melt-extruded solid dispersions,^20,21^ and the total solubility parameter of 24 MPa^0.5^ was inferred from the literature.^48^ Accordingly, any excipient mixed with cinnarizine should have a total solubility parameter within 9–29 MPa^0.5^, while for HPC, the interval was 14–34 MPa^0.5^, giving overlapping values at 14–29 MPa^0.5^ as ORSP. Alternatively using the more restrictive solubility parameter interval of ±7 MPa^0.5^, the ORSP yields 17–26 MPa^0.5^. As for bifonazole, the broader and hence more inclusive solubility parameter interval provides an ORSP of 14–30 MPa^0.5^, whereas the more restrictive ORSP yields 17–27 MPa^0.5^. In the next step, a series of 34 small-molecular additives were selected based on oral acceptability and with the requirement that the calculated total solubility parameter was within the relatively broader limits of the ORSP (Figure 1). Although, in principle, the ORSP is drug-specific, the high similarity in the case of the selected model drugs led to the same selection of excipient candidates. For further selection of most promising coformers, the excess enthalpy, *H*_ex_, was calculated in the framework of COSMO-RS theory.^35^ A fragment-based approach was employed, which makes use of a database of molecular screening charge distributions obtained from previous quantum chemical calculations.^46^ The estimated excess enthalpy has been used before to screen excipients regarding formation of cocrystals with the drug^35,36^ or successful coamorphous mixtures.^37^ As the present work focused on hot melt-extruded solid dispersions, a new approach was to map *H*_ex_ values of the excipient candidates with the drug for a suitable temperature process range of the given polymer. A high temperature value close to the thermal decomposition temperature of the polymer may be selected, or as in the present case, a more conservative upper limit (i.e., 225 °C). The lower temperature limit can be selected based on glass transition temperature and extrusion experience with the given polymer; here, a value of 100 °C was taken for HPC.^53^ As a result, Figure 2 displays the mapping of *H*_ex_ of the drug and excipient vs their melting points. Several additives were outside the targeted process range for hot melt extrusion of HPC. With a lower temperature range, it makes sense to avoid additives that are either liquid or waxy, which in larger quantities are difficult to dispense into the extruder barrel. Moreover, for compounds with high melting points outside the process temperature range, drug interactions would only be possible on the surface of suspended additive particles instead of the desirable molecular-level mixture of the components. As shown in Figure 2, the most pronounced interactions were found for a group of three acids in order of increasing melting points: malic, citric, and tartaric acids. The fact that the same group of three acids had the highest interaction potential for both drugs indicates that some similarity exists between cinnarizine and bifonazole, which was already evident from the very similar total solubility parameters. However, there were also differences noted in the predicted *H*_ex_ values in that the interactions of excipients and bifonazole reached clearly more negative values than the corresponding excipients with cinnarizine. A slightly negative value of *H*_ex_ may already result in an amorphous product, as there is further contributing mixing entropy for the relevant free energy of mixing. More promising are certainly highly negative *H*_ex_ values whereby more negative values beyond −2 kcal/mol generally resulted in amorphous products as suggested by a recent work on coamorphous binary systems.^38^ Slightly negative *H*_ex_ values were shown to still result in amorphous 1:1 products in the case that the coformer and drug had similar lipophilicity.^38^ Great differences in lipophilicity would be reflected in mismatches of the total solubility parameter, where the latter parameter should be the more relevant characteristic than lipophilicity regarding miscibility and possibly also for physical stability. As the present work used only preselected coformers based on the ORSP, the slightly negative *H*_ex_ values in the case of cinnarizine and selected coformers still hold promise for a subsequent experimental study of the ternary ASDs.

While malic, citric, and tartaric acids were identified as preferred additives based on negative *H*_ex_ values, a clearly unfavorable excipient was ornithine as it showed even positive excess enthalpy with both drugs and was accordingly selected as a negative control additive.

**Figure 3:**
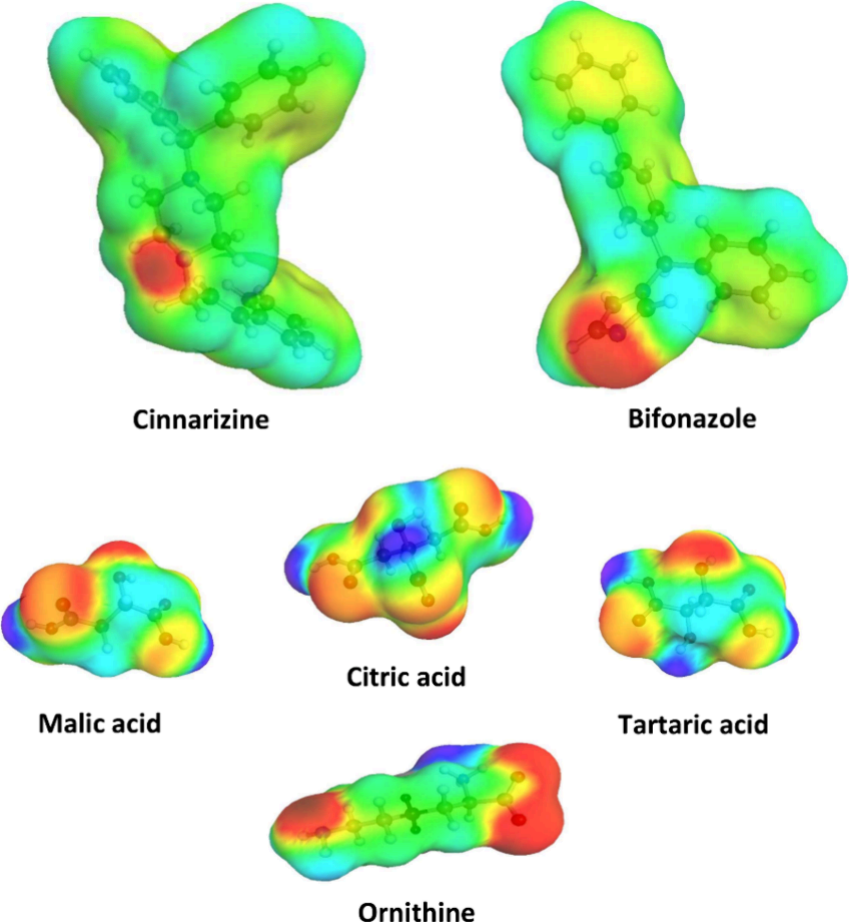
Sigma surfaces obtained from density functional theory (DFT) are shown for the model drugs and selected coformers. Low to high screening charge is color-coded from blue to red with neutral surfaces in green.

Figure 3 shows the so-called sigma surface, which depicts the surface screening charges from the DFT calculations. There were similarities noted between the model drugs regarding a high fraction of practically neutral (in green) surface charge and with respect to distinct basicity, as shown by the highly positive screening charge of the respective nitrogen atoms (in red) corresponding to high electron density. The coformers malic, citric, and tartaric acids shared together with ornithine the similarity that all molecules were comparatively small and showed hydrogen bonding as well as accepting qualities, but there were still individual differences in geometry and extent of very low and high screening charges on the molecular surfaces.

### 3.2 Experimental Feasibility Testing Using Hot Melt Extrusion

**Figure 4:**
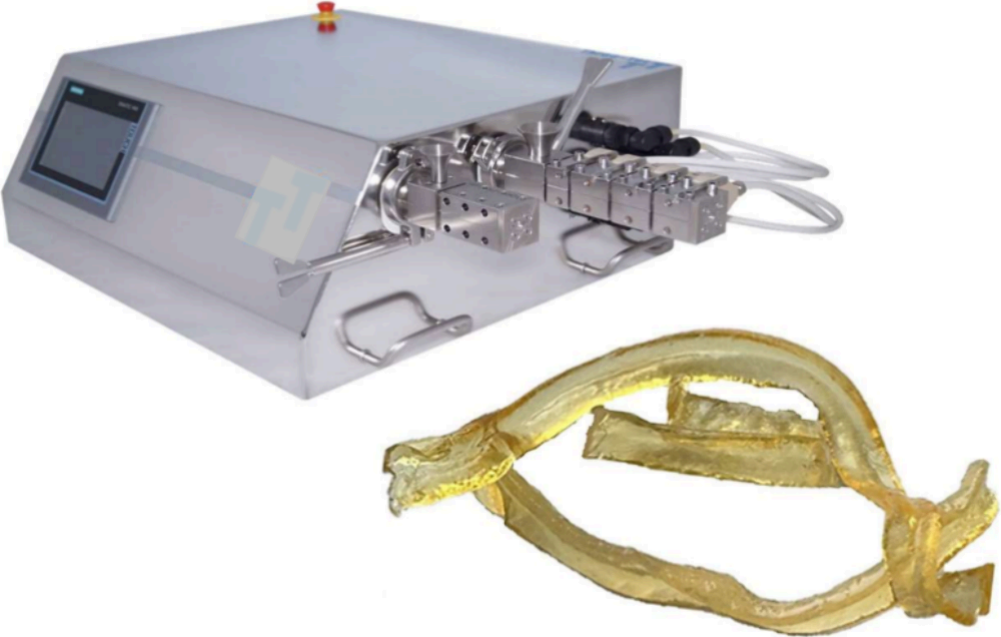
Small-scale hot melt extruder (twin screw, 9 mm, ZE9 ECO by ThreeTec Ltd.) and extrudates of a solid dispersion using HPC SL and malic acid.

Before embarking on more formal stability testing, some technical feasibility trials were conducted on small-scale manufacturing equipment (Figure 4) by considering different HPC grades. Thus, first extrusion experiments used a process temperature of 150 °C for pure polymer of grade SL (∼100 kDa) and grade SSL (∼40 kDa), which in both cases resulted in extrudates with a smooth and mostly transparent appearance. The HPC L grade (∼140 kDa) required a higher extrusion temperature of around 180 °C, and the extrudate appearance obtained was like the other grades, although transparency appeared to be lower and the yellowish color of the HPC extrudates was slightly darker. The good technical feasibility of HPC grades observed in hot melt extrusion confirmed results of earlier studies, and the pioneering work of Sarode et al.^54^ is worth noting. Given the previously measured melting points of 120.6 °C for cinnarizine and 149.4 °C for bifonazole,^11^ it would have been possible to select any of the tested HPC grades. The SL grade was arbitrarily selected, as its molecular weight was between the SSL and L grades.

As the computational screening focused on citric, malic, and tartaric acids, each of these coforming excipients was first explored for their technical processing feasibility at 20% (w/w) additive with 5% (w/w) of API and HPC SL as a polymeric matrix. The rationale for the compositional selection was that a slight excess of the coformer relative to the drug was targeted with a sufficient polymer matrix to enable an extrusion process. Moreover, the initial feasibility trials used a lower drug concentration than 15% (w/w) in the subsequent stability tests for a screening purpose.

The process temperature was normally 150 °C but was increased by at least 15 °C in the case of tartaric acid mixtures because of the slightly higher melting point of this excipient. The processability of the different mixtures was generally good, and slightly yellow extrudates were obtained with different levels of transparency. Extrudates with citric acid displayed a tendency to become brittle, and although this technical issue could be tackled by adding a plasticizer, the scope of the present research was to keep the formulation simple with only one polymer and coforming additive. Another aspect was that citric acid had been shown to degrade at 168 °C,^55^ so there is a risk of initial degradation even at the lower process temperature selected. Accordingly, the main stability experiments focused only on malic and tartaric acids as preferred additives. Moreover, ornithine was further included as a negative control additive for which the calculated *H*_ex_ value was less favorable in the case of both model drugs (Figure 2). The corresponding hot melt extrusion of ornithine at 20% (w/w) in HPC SL demonstrated good processability. The extrudate appearance was again slightly yellow, and some cloudiness was noted. However, slight turbidity is rather unspecific, and further bulk analytics would be needed to interpret such a visual appearance, which was the scope of the subsequent stability trials.

### 3.3 Physical Stability of Candidate Formulations

Following computational excipient screening and initial melt extrusion feasibility trials, the selected formulations were manufactured and physically analyzed. Apart from common bulk analytics using DSC and XRPD, the extrudates were also studied by means of confocal laser scanning and AFM imaging. Nanoimaging has been identified as a sensitive analytical method, either to detect even small amounts of residual crystallinity following manufacture or to detect early phase separation before physical instability may be found by means of conventional DSC or XRPD.^42^ The need for orthogonal solid dispersion analytics has been emphasized by current Taylor group research, which suggested that even low levels of drug crystallinity below the detection limit of conventional thermal analysis or X-ray diffraction can impact phase behavior on release.^56^ Pioneering work on confocal laser scanning microscopy in solid dispersion analytics has been published in the past few years,^57^ and an advantage over AFM imaging is the broader field of view in which crystals can be sampled. Therefore, it is beneficial to combine confocal microscopy with AFM as the former can serve as a screening tool for crystal detection, while the latter’s more sensitive spatial resolution may detect sensitively a beginning phase separation. Such combined imaging methods have been reported to detect early signs of physical instability in amorphous solid dispersions before it became apparent using DSC of XRPD analysis.^42^ As the use of the latter methods is standard in the solid-state analysis of amorphous formulations, these techniques are also combined with the imaging methods in the present study.

**Figure 5:**
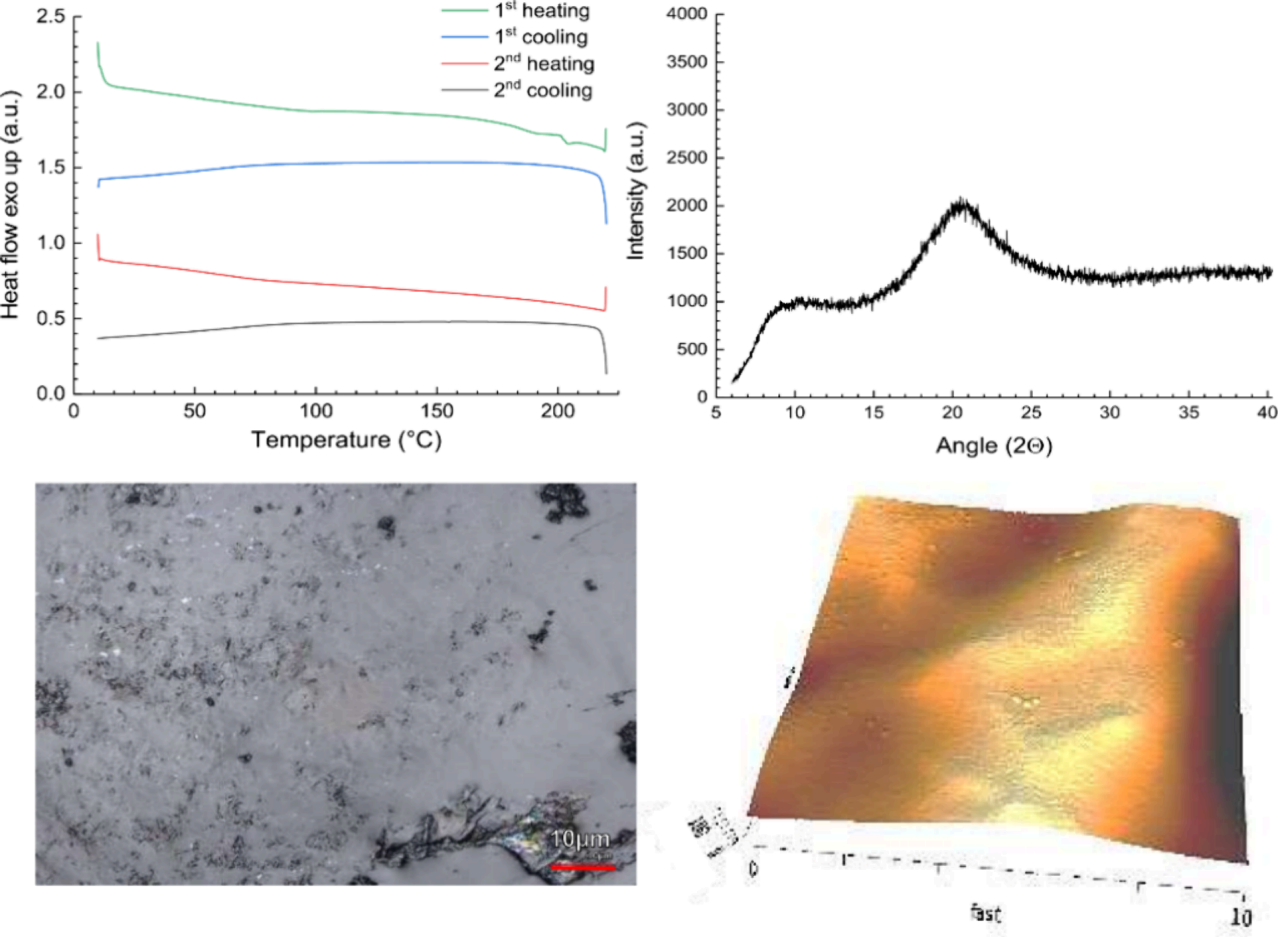
Example of a completely amorphous system (e.g., the binary mixtures of HPC SL and malic acid) as analyzed by differential scanning calorimetry (DSC), X-ray powder diffractometry (XRPD), confocal laser scanning microscopy, and atomic force microscopy (AFM) (after 3 months of storage at RT, sealed).

The blends of the polymer with the coformer were extruded and analyzed; results can be inferred from Table 1. The use of the preferred coformers malic and tartaric acids both resulted in completely amorphous systems based on DSC and XRPD analysis. The more sensitive imaging techniques confirmed the homogeneous amorphous state of the formulation with malic acid (Figure 5), while some small needles were detected on the surface of the extrudates in the case of tartaric acid. These needles had the identical habitus as pure tartaric acid; it is possible that these crystalline traces below the limit of DSC and XRPD detection reflected a surface phenomenon. These traces of crystallinity appeared to be unchanged in extent over the stability period, so there was no progressive crystallization noted that could become detectable by bulk analytics.^58^

**Table 1:** Overview of Physical Characterization from Binary Mixtures of Polymer and Additives

HPC SL in binary mixtures	DSC & XRPD	laser scanning & AFM imaging
dl-malic acid (20%, w/w)	amorphous with both analytical methods (initial and 3 M data)	absence of any crystalline material initially and after 3 months
l-ornithine (20%, w/w)	DSC amorphous but small amounts of crystalline ornithine in XRPD (initial and 3 M analysis)	crystals found in the samples have the same habitus as ornithine (initial and 3 M)
l-tartaric acid (20%, w/w)	amorphous with both analytical methods (initial and 3 M data)	amorphous samples but very few small needles on the surface detected like those of pure tartaric acid

Based on the DSC thermogram, the negative control additive coformer ornithine was only partially amorphous as it displayed at the initial time point some Bragg peaks in the XRPD, primarily at 8.8, 20.8, 23.6, 23.8, and 29.0° (2θ), which corresponded to the diffractogram taken from pure ornithine. A partially amorphous matrix was also supported by laser scanning and AFM imaging. It is worth noting that the solubility parameter of ornithine was close to the lower limit of the broader ORSP, while that of the preferred coformers malic and tartaric acids was toward the center of the range and hence close to that of HPC (Figure 1). The defined ranges of ±10 MPa were taken from the literature regarding likely demixing outside this solubility parameter interval, which is different from expected full miscibility for the more restrictive interval of ±7 MPa.^51^Figure 1 shows that ornithine was indeed outside of the more restrictive ORSP. The broad range of ±10 MPa still appears reasonable for multicomponent mixtures, as ORSP considerations were followed by more advanced COSMO-RS modeling. Finally, an added drug can also influence coformer miscibility in the matrix, especially when strong molecular interactions are targeted. It finally remains a strategic decision in early formulation screening if the ORSP should be used with the more restrictive or otherwise more inclusive solubility parameter interval (Figure 1).

**Figure 6:**
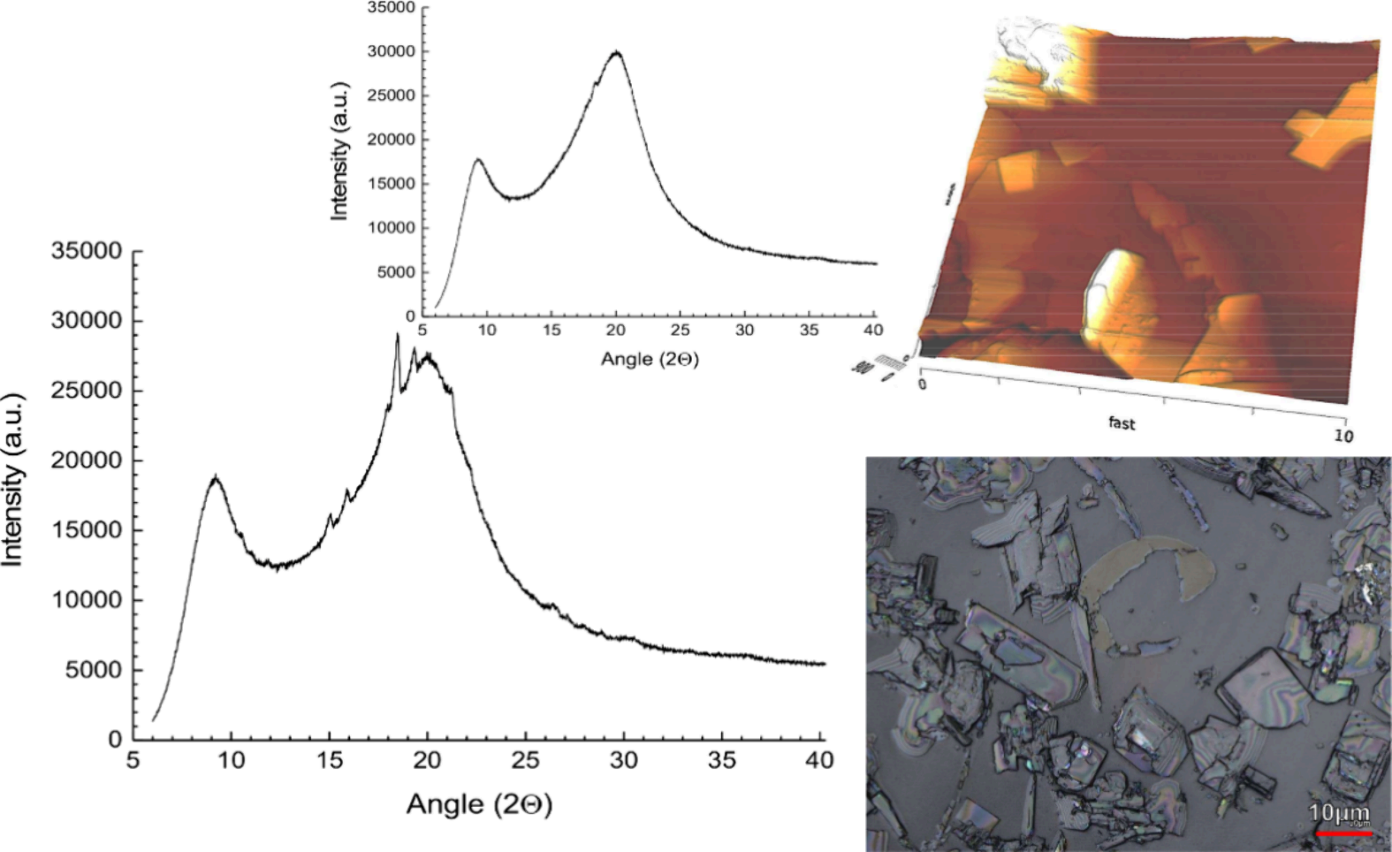
Binary solid dispersion of bifonazole and HPC SL grade as an initial XRPD diffractogram (inset) and after 3 months of storage (RT, sealed), together with confocal laser scanning microscopy and AFM imaging.

Next, extrudates of the model compound bifonazole were prepared; an overview of the extruded products’ physical studies is given in Table 2. Bifonazole has been previously reported as an unstable glass-forming compound.^11^ Some previous work did not even attempt to obtain a fully amorphous product and focused rather on crystalline solid dispersions of bifonazole while keeping the particle size small.^59^ The present result of the binary solid dispersion bifonazole in combination with HPC SL alone showed an initial DSC and XRPD analysis that suggested a fully amorphous solid dispersion. The absence of crystals was also confirmed by confocal laser scanning and AFM analysis. After 3 months, the DSC of the extrudates did not give a clear endotherm, suggesting crystallization of bifonazole, but the diffractogram showed small peaks above the amorphous halo (Figure 6). The Bragg peaks between 15 and 19° (2θ) corresponded to those of the pure drug diffractogram; this agreed with laser scanning as well as with AFM images, in which platelets of the crystalline drug were identified following the storage time (3 M, 25 °C). Therefore, HPC alone as a polymer matrix was insufficient to stabilize the binary solid dispersions of bifonazole, which stresses the need for harnessing strong molecular interactions with a coformer to achieve better physical stability.

**Table 2:** Overview of Physical Characterization from Solid Dispersions including Bifonazole

HPC SL in binary or ternary mixtures	DSC & XRPD	laser scanning & AFM imaging
bifonazole (15%, w/w)	initial analysis suggested an amorphous product, but following storage (3 M, 25 °C), small Bragg peaks were observed	crystalline platelets of drug were revealed by both imaging methods but only following storage (3 M, 25 °C)
bifonazole (15%, w/w), dl-malic acid (20%, w/w)	DSC and XRPD indicated an amorphous mixture of initial and stored samples	both imaging techniques suggested the absence of crystals
bifonazole (15%, w/w), l-ornithine (20%, w/w)	DSC only detected crystallinity after storage using powdered samples; XRPD already demonstrated a partially crystalline mixture following preparation	both imaging techniques demonstrated partial crystallinity, although distinction of the coformer and drug was difficult
bifonazole (15%, w/w), l-tartaric acid (20%, w/w)	DSC and XRPD also suggested an amorphous mixture after storage	some crystals were detected that were assigned to tartaric acid but not the drug

Based on DSC and XRPD analysis, the ternary mixture of HPC SL with malic acid and bifonazole was amorphous, both on initial analysis as well as following 3 months of stability testing. Moreover, no crystals were found by the confocal laser scanning or in the AFM images. Thus, the thermograms, diffractograms, and images looked essentially the same as those for the binary mixture of polymer and malic acid (Figure 5). It was notable that the challenging bifonazole was evidently stable in this ternary system, whereas this was not the case for the binary HPC SL and bifonazole solid dispersion, as outlined in Figure 6. As predicted by the excess enthalpy calculations of the coformer and drug, malic acid proved to be an excellent stabilizing additive. The arbitrarily selected concentrations targeted a slight excess of the coformer compared to the drug in a formulation, unlike classical coamorphous systems that are preferably formulated at the 1:1 molar ratio or that of their corresponding eutectic mixture.^60^ Another difference of ternary solid dispersions compared with coamorphous systems is that a polymer is rarely added and, if so, only in a small amount of 5–10% (w/w).^28^ These compositional differences are also reflected in possible process technologies, as ternary solid dispersions can be obtained by hot melt extrusion, which enables continuous manufacturing with a low production footprint.^5^

The ternary system of HPC SL, bifonazole, and the negative control coformer ornithine already exhibited partial crystallinity in the initial samples by means of XRPD and imaging. Most of the crystals identified in the confocal laser scanning images could be assigned to the habitus of ornithine, and also XRPD showed the aforementioned Bragg peaks of the coformer, while it remained not clear if there was a small peak of bifonazole, which showed as pure material most predominant peaks at 10.5, 11.9, and 21.2° (2θ). Further samples were stored under ambient conditions (i.e., not sealed in aluminum bags) and analyzed using confocal laser scanning and AFM; in those samples, it was possible to detect small plates corresponding to the typical habitus of bifonazole. The results obtained for these ternary mixtures suggested the possible crystallinity of bifonazole, although this was difficult to demonstrate clearly given that ornithine had apparently crystallized to some extent. In any case, and as expected based on the prior *in silico* evaluation, the ternary mixture was not pharmaceutically favorable for the negative control additive of ornithine.

**Figure 7:**
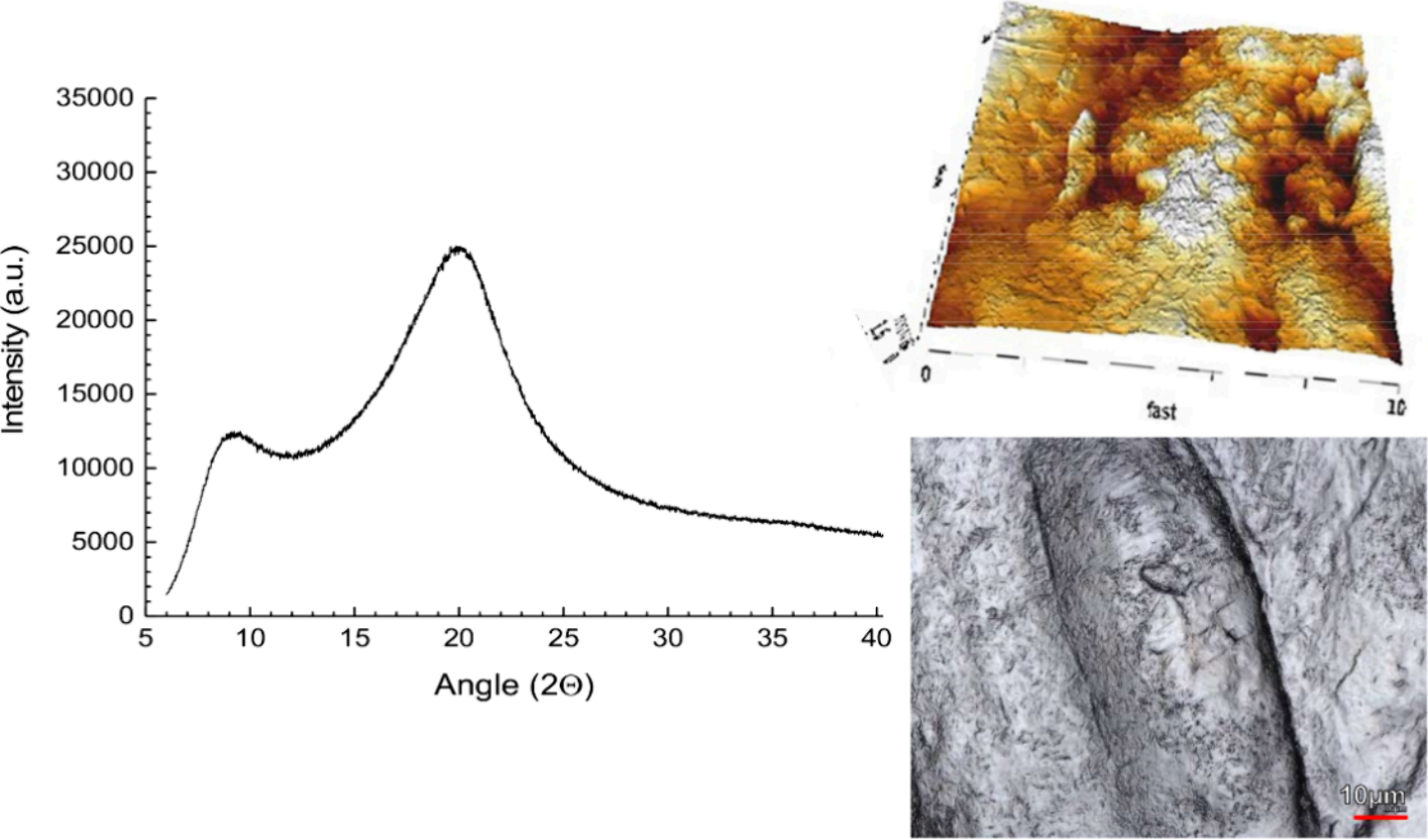
Ternary solid dispersion of bifonazole, tartaric acid, and HPC SL grade as an XRPD diffractogram together with corresponding laser scanning imaging and AFM imaging after 3 months of storage (RT, sealed).

The formulation of HPC SL, bifonazole, and the tartaric acid coformer was similar to that with malic acid, in that no crystallinity was detected by means of conventional bulk analytics, either in initial samples or those stored for 3 months (Table 2 and Figure 7). Imaging suggested that some crystals already existed in the initial samples, but even in the stored samples, no crystals could be assigned to the drug as they all showed the typical small needle shape of tartaric acid. Therefore, malic and tartaric acids, selected based on COSMO-RS calculations, also provided experimentally stable amorphous mixtures at least during the study duration. This was an encouraging result given prior efforts to stabilize the unstable drug bifonazole in an amorphous solid dispersion. The experimental findings of bifonazole solid dispersions support the view that the use of small-molecular additives combined with a suitable polymer can be beneficial for the physical stability of solid dispersions. Unstable glass-forming compounds pose a substantial formulation challenge for amorphous drug products; the digital approach presented here did indeed lead to promising candidate formulations of ternary solid dispersions of bifonazole.

**Figure 8:**
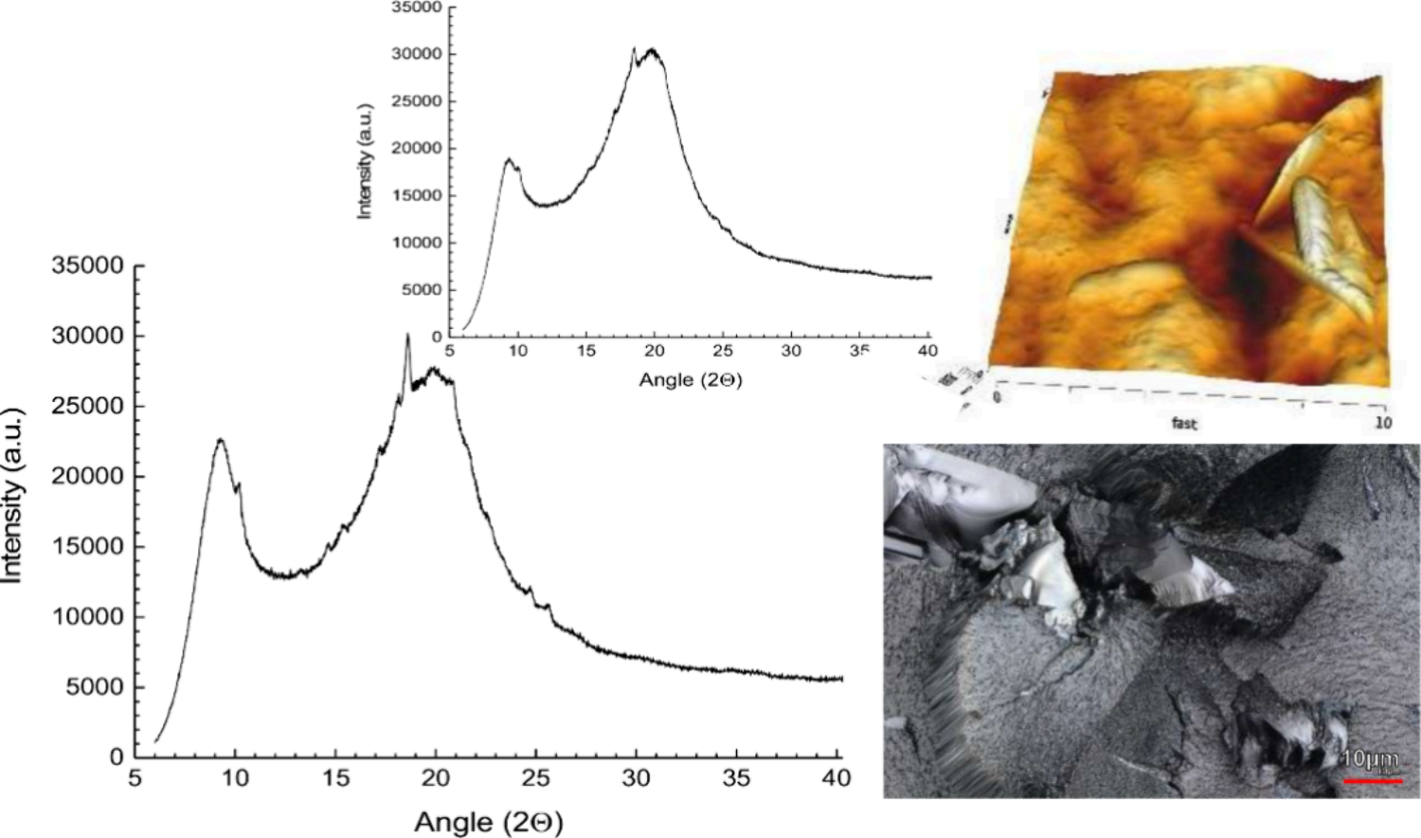
Binary solid dispersion of cinnarizine and HPC SL grade as an initial XRPD diffractogram (inset) and after 3 months of storage (RT, sealed) together with corresponding laser imaging and AFM imaging.

In line with the study aims, cinnarizine was selected as a further model compound for an unstable glass-forming drug (class II). As with bifonazole and based on total solubility parameters, HPC was a suitable polymer selection for this drug. However, this would mean only the expected miscibility of the components. Lack of miscibility is a known indicator of poor stability, as separation into drug-poor and drug-rich polymer phases would lead to increased crystallization in the latter.^61^ Thus, while the good miscibility of the formulation components is generally targeted in solid dispersions, crystallization is still often encountered, especially over time in the case of unstable glass-forming compounds.^62^ Earlier work on cinnarizine found that there was comparatively low activation energy for the amorphous drug crystallization kinetics.^6^ A rather high level of interaction with the matrix would therefore be needed to overcome this inherent drug instability. Table 3 shows that even the samples after hot melt extrusion showed small amounts of crystallinity in DSC as well as XRPD. The latter revealed a small initial peak at around 18.6° (2θ) (Figure 8), corresponding to the most pronounced Bragg signal of pure reference cinnarizine. The latter pure cinnarizine reference showed further weaker XRPD at 18.0, 17.7, 10.1, 20.8, 13.2, 21.7, and 22.6° (2θ*)*, here listed in order of declining peak height. Figure 8 reveals that after storage, not only did the 18.6° (2θ) peak grow slightly but also further minor peaks appeared in these aged samples in line with what could be expected for crystalline cinnarizine. The confocal laser imaging showed many of the typical needle-shaped crystals, and even in the AFM images, the needle-like protrusions were individually visible.

**Table 3:** Overview of Physical Characterization from Solid Dispersions including Cinnarizine

HPC SL in binary or ternary mixtures	DSC & XRPD	laser scanning & AFM imaging
cinnarizine (15%, w/w)	initial DSC thermogram and XRPD both suggested minor crystallinity, initially and more pronounced after storage (3 M, 25 °C)	Increasing crystallinity over storage time was supported by imaging, with visible needles in confocal laser scanning and typical long protrusions in AFM
cinnarizine (15%, w/w), dl-malic acid (20%, w/w)	DSC and XRPD showed no trace of crystallinity, either in the initial sample nor after storage (3 M, 25 °C)	laser confocal scanning and AFM imaging support the view of an amorphous mixture, both initially and after storage (3 M, 25 °C)
cinnarizine (15%, w/w), l-ornithine (20%, w/w)	drug-related Bragg peaks were clearly visible and grew over the 3 month storage time; DSC did not show a clearly visible melt endotherm after storage (3 M, 25 °C)	laser microscopy and AFM imaging suggested partially crystalline samples at time zero and after storage (3 M, 25 °C)
cinnarizine (15%, w/w), l-tartaric acid (20%, w/w)	DSC and XRPD did not show signs of crystallinity after sample preparation or storage (3 M, 25 °C)	confocal scanning and AFM with the different samples showed individual crystals smaller than the typical cinnarizine needles

**Figure 9:**
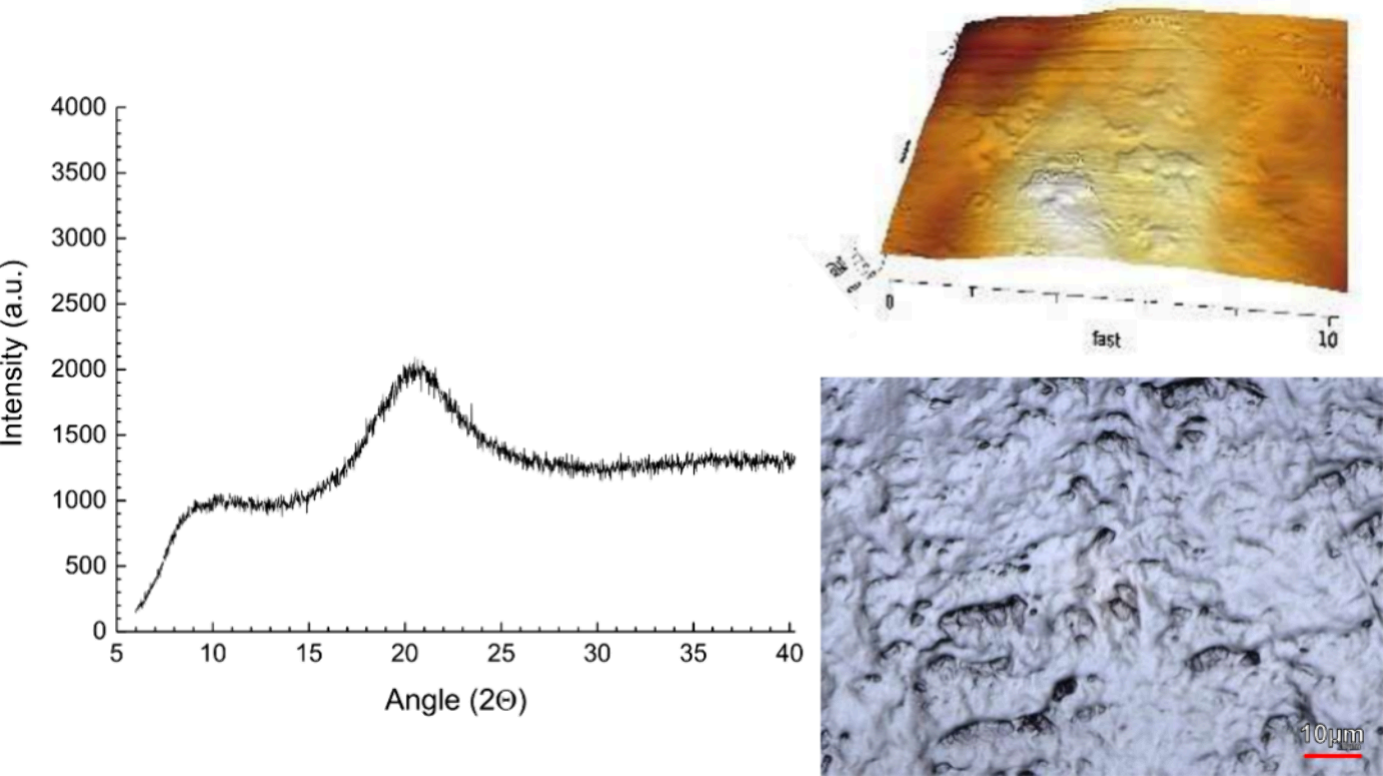
Ternary solid dispersion of cinnarizine, malic acid, and HPC SL grade as an XRPD diffractogram together with corresponding laser imaging and AFM imaging after 3 months of storage (RT, sealed).

It was subsequently of interest whether the digitally selected additives could improve the physical stability as a ternary ASD. Interestingly, malic acid at 20% (w/w) was successful regarding the amorphous dispersion formulation in the initial analysis (Table 3). Even the sensitive imaging techniques did not show the typical cinnarizine crystals, either initially or after 3 months of storage (Figure 9). This positive result contrasted with the experiments with the negative control additive ornithine. In line with the previous findings of the binary additive and HPC mixtures, much crystalline ornithine was already present at the initial time point. XRPD showed the dominant Bragg peaks of ornithine, but the 18.6° (2θ) of cinnarizine was also visible, suggesting that not only the coformer crystallized. This finding of a mixed crystalline sample of the additive and drug was also supported by the two imaging techniques in which many crystals were identified. Following these negative control ornithine mixtures, it was then of interest to learn whether tartaric acid would successfully lead to a completely amorphous sample. The tartaric acid solid dispersions were indeed amorphous based on DSC and XRPD analysis, while the more sensitive imaging techniques spotted individual crystals on the surface. These were small needles and, therefore, they did not resemble the habitus of pure cinnarizine crystals but rather looked like tartaric acid as seen from reference crystals. Such an occurrence of few coformer crystals could have been due to surface crystallization in the presence of moisture, as on a bulk level, the samples appeared to be homogeneous with the amorphous drug even after storage.

The present finding of successful ternary solid dispersion formation of cinnarizine can be compared to another recent study on polymer-based solid dispersions of the same active compound.^23^ This previous work was on cinnarizine-soluplus solid dispersions, as well as on the addition of the polymers hydroxypropylmethyl cellulose (HPMC) and polyvinylpyrrolidone (PVP), but also studied the small-molecular additives sorbitol and citric acid. The selection of an additional polymer to optimize drug release has often been tried, and such additional excipients have been found to affect drug stability.^18^ In the case of small-molecular additives, they can exert a kind of plasticizer or antiplasticizer effect according to their melting points and in line with the Gordon–Taylor equation.^63^ Such additive selection is primarily aimed at local molecular mobility and, hence, kinetic stabilization of amorphous drugs.^64^ The aforementioned cinnarizine-soluplus solid dispersion study is interesting, as the authors also tested sorbitol and citric acid in solid dispersions with the same rationale.^23^ While no stabilizing effect was observed for sorbitol, the addition of citric acid had a clearly stabilizing effect on cinnarizine. The authors argued that citric acid may act as a kind of molecular link between the polymer and cinnarizine.^23^ However, the present COSMO-RS study identified citric, tartaric, and malic acids as possible conformers with the highest negative excess enthalpy values in combination with the model drugs. Therefore, such conformers would interact primarily as drug complexing agents and perhaps also in configurations corresponding to the above-mentioned drug-linking with the polymer.

The main progress achieved by the present study is that identification of a stabilizing additive should no longer be guided by serendipity or the assumption of an antiplasticizing effect but rather via a computational approach. The ORSP considerations presented, followed by mapping of excess enthalpy values by COSMO-RS, are a way forward to avoid a growing number of experiments in line with the need for more complex solid dispersion formulations.

## 4 Conclusions

While unstable glass-forming compounds have remained a challenge to formulate as amorphous solid dispersions, ternary solid dispersions appear to be a versatile and highly promising way to cope with the inherent tendency of challenging compounds to recrystallize. Although sufficient kinetic stability is dependent on a variety of factors, a particularly strong molecular interaction with a coformer and, possibly, the polymer matrix is certainly one of the most attractive strategies. Targeting such strong specific interactions would be highly resource-intensive if it relied exclusively on experimentation; therefore, *in silico* screening must be a part of such a formulation strategy. The proposed new approach, using ORSP for subsequent mapping of melting points and calculating excess enthalpy values of the coformer and drug, was found to be useful for identifying preferred coformer mixtures. Future work may expand on the complexity of the COSMO-RS model by taking humidity into account, and a biopharmaceutical study can follow up on the present research on physical stability. The candidate formulations are expected to have further advantages regarding drug release, given the relatively high solubility of the conformers and possible interactions with both the polymer and coformer, which can stabilize drug supersaturation. Such biopharmaceutical work was beyond the scope of the present study on stability but can be part of future research. More research is expected over the coming years focusing on *in silico* tools for finding more complex solid dispersions or bioenabling formulations in general.

## Data Availability

Research data used in preparation of the manuscript can be obtained from the corresponding author upon request.

## References

[ref1] Butler J. M.; Dressman J. B. The Developability Classification System: Application of Biopharmaceutics Concepts to Formulation Development. J. Pharm. Sci. 2010, 99 (12), 4940–4954. 10.1002/jps.22217. 20821390

[ref2] Buckley S. T.; Frank K. J.; Fricker G.; Brandl M. Biopharmaceutical classification of poorly soluble drugs with respect to ″enabling formulations″. European Journal of Pharmaceutical Sciences 2013, 50 (1), 8–16. 10.1016/j.ejps.2013.04.002. 23583787

[ref3] Kuentz M.; Holm R.; Kronseder C.; Saal C.; Griffin B. T. Rational Selection of Bio-Enabling Oral Drug Formulations-A PEARRL Commentary. J. Pharm. Sci. 2021, 110 (5), 1921–1930. 10.1016/j.xphs.2021.02.004. 33609523

[ref4] Vo C. L. N.; Park C.; Lee B. J. Current trends and future perspectives of solid dispersions containing poorly water-soluble drugs. Eur. J. Pharm. Biopharm. 2013, 85 (3), 799–813. 10.1016/j.ejpb.2013.09.007. 24056053

[ref5] Alzahrani A.; Nyavanandi D.; Mandati P.; Youssef A. A. A.; Narala S.; Bandari S.; Repka M. A systematic and robust assessment of hot-melt extrusion-based amorphous solid dispersions: Theoretical prediction to practical implementation. Int. J. Pharm. 2022, 624, 121951 10.1016/j.ijpharm.2022.121951. 35753536

[ref6] Baghel S.; Cathcart H.; O’Reilly N. J. Polymeric Amorphous Solid Dispersions: A Review of Amorphization, Crystallization, Stabilization, Solid-State Characterization, and Aqueous Solubilization of Biopharmaceutical Classification System Class II Drugs. J. Pharm. Sci. 2016, 105 (9), 2527–2544. 10.1016/j.xphs.2015.10.008. 26886314

[ref7] Kawakami K. Theory and practice of supersaturatable formulations for poorly soluble drugs. Therapeutic delivery 2015, 6 (3), 339–352. 10.4155/tde.14.116. 25853309

[ref8] Boyd B.; Bergstrom C. A. S.; Vinarov Z.; Kuentz M.; Brouwers J.; Augustijns P.; Brandl M.; Bernkop-Schnurch A.; Shrestha N.; Preat V.; et al. Successful oral delivery of poorly water-soluble drugs both depends on the intraluminal behavior of drugs and of appropriate advanced drug delivery systems. European Journal of Pharmaceutical Sciences 2019, 137, 104967 10.1016/j.ejps.2019.104967. 31252052

[ref9] Baird J. A.; Van Eerdenbrugh B.; Taylor L. S. A Classification System to Assess the Crystallization Tendency of Organic Molecules from Undercooled Melts. J. Pharm. Sci. 2010, 99 (9), 3787–3806. 10.1002/jps.22197. 20623696

[ref10] Alhalaweh A.; Alzghoul A.; Kaialy W.; Mahlin D.; Bergstrom C. A. S. Computational Predictions of Glass-Forming Ability and Crystallization Tendency of Drug Molecules. Mol. Pharmaceutics 2014, 11 (9), 3123–3132. 10.1021/mp500303a. 25014125

[ref11] Wyttenbach N.; Kirchmeyer W.; Alsenz J.; Kuentz M. Theoretical Considerations of the Prigogine-Defay Ratio with Regard to the Glass-Forming Ability of Drugs from Undercooled Melts. Mol. Pharmaceutics 2016, 13 (1), 241–250. 10.1021/acs.molpharmaceut.5b00688. 26587865

[ref12] Blaabjerg L. I.; Lindenberg E.; Rades T.; Grohganz H.; Löbmann K. Influence of preparation pathway on the glass forming ability. Int. J. Pharm. 2017, 521 (1–2), 232–238. 10.1016/j.ijpharm.2017.02.042. 28232267

[ref13] Alhalaweh A.; Alzghoul A.; Mahlin D.; Bergstrom C. A. S. Physical stability of drugs after storage above and below the glass transition temperature: Relationship to glass-forming ability. Int. J. Pharm. 2015, 495 (1), 312–317. 10.1016/j.ijpharm.2015.08.101. 26341321 PMC4622963

[ref14] Blaabjerg L. I.; Bulduk B.; Lindenberg E.; Löbmann K.; Rades T.; Grohganz H. Influence of Glass Forming Ability on the Physical Stability of Supersaturated Amorphous Solid Dispersions. J. Pharm. Sci. 2019, 108 (8), 2561–2569. 10.1016/j.xphs.2019.02.028. 30878513

[ref15] Wyttenbach N.; Kuentz M. Glass-forming ability of compounds in marketed amorphous drug products. Eur. J. Pharm. Biopharm. 2017, 112, 204–208. 10.1016/j.ejpb.2016.11.031. 27903457

[ref16] Kawakami K.; Usui T.; Hattori M. Understanding the glass-forming ability of active pharmaceutical ingredients for designing supersaturating dosage forms. J. Pharm. Sci. 2012, 101 (9), 3239–3248. 10.1002/jps.23166. 22531946

[ref17] Ditzinger F.; Price D. J.; Ilie A.-R.; Kohl N. J.; Jankovic S.; Tsakiridou G.; Aleandri S.; Kalantzi L.; Holm R.; Nair A.; et al. Lipophilicity and hydrophobicity considerations in bio-enabling oral formulations approaches - a PEARRL review. J. Pharm. Pharmacol. 2019, 71 (4), 464–482. 10.1111/jphp.12984. 30070363

[ref18] Al-Obaidi H.; Ke P.; Brocchini S.; Buckton G. Characterization and stability of ternary solid dispersions with PVP and PHPMA. Int. J. Pharm. 2011, 419 (1–2), 20–27. 10.1016/j.ijpharm.2011.06.052. 21801822

[ref19] Prasad D.; Chauhan H.; Atef E. Amorphous Stabilization and Dissolution Enhancement of Amorphous Ternary Solid Dispersions: Combination of Polymers Showing Drug-Polymer Interaction for Synergistic Effects. J. Pharm. Sci. 2014, 103 (11), 3511–3523. 10.1002/jps.24137. 25196860

[ref20] Bachmaier R. D.; Monschke M.; Faber T.; Krome A. K.; Pellequer Y.; Stoyanov E.; Lamprecht A.; Wagner K. G. In vitro and in vivo assessment of hydroxypropyl cellulose as functional additive for enabling formulations containing itraconazole. International journal of pharmaceutics: X 2021, 3, 100076–100076. 10.1016/j.ijpx.2021.100076. 33851133 PMC8024662

[ref21] Niederquell A.; Stoyanov E.; Kuentz M. Hydroxypropyl Cellulose for Drug Precipitation Inhibition: From the Potential of Molecular Interactions to Performance Considering Microrheology. Mol. Pharmaceutics 2022, 19 (2), 690–703. 10.1021/acs.molpharmaceut.1c00832. 35005970

[ref22] Borde S.; Paul S. K.; Chauhan H. Ternary solid dispersions: classification and formulation considerations. Drug Dev. Ind. Pharm. 2021, 47 (7), 1011–1028. 10.1080/03639045.2021.1908342. 33818224

[ref23] Tian B.; Ju X. K.; Yang D.; Kong Y.; Tang X. Effect of the third component on the aging and crystallization of cinnarizine-soluplus® binary solid dispersion. Int. J. Pharm. 2020, 580, 119240 10.1016/j.ijpharm.2020.119240. 32197983

[ref24] Dengale S. J.; Grohganz H.; Rades T.; Löbmann K. Recent advances in co-amorphous drug formulations. Adv. Drug Delivery Rev. 2016, 100, 116–125. 10.1016/j.addr.2015.12.009. 26805787

[ref25] Karagianni A.; Kachrimanis K.; Nikolakakis I. Co-Amorphous Solid Dispersions for Solubility and Absorption Improvement of Drugs: Composition, Preparation, Characterization and Formulations for Oral Delivery. Pharmaceutics 2018, 10 ( (3), ). DOI: 98 10.3390/pharmaceutics10030098. 30029516 PMC6161132

[ref26] Ueda K.; Moseson D. E.; Pathak V.; Taylor L. S. Effect of Polymer Species on Maximum Aqueous Phase Supersaturation Revealed by Quantitative Nuclear Magnetic Resonance Spectroscopy. Mol. Pharmaceutics 2021, 18 (3), 1344–1355. 10.1021/acs.molpharmaceut.0c01174. 33595322

[ref27] Price D. J.; Ditzinger F.; Kohl N. J.; Jankovic S.; Tsakiridou G.; Nair A.; Holm R.; Kuentz M.; Dressman J. B.; Saal C. Approaches to increase mechanistic understanding and aid in the selection of precipitation inhibitors for supersaturating formulations - a PEARRL review. J. Pharm. Pharmacol. 2019, 71 (4), 483–509. 10.1111/jphp.12927. 29770440

[ref28] Wang Y. X.; Grohganz H.; Rades T. Effects of polymer addition on the non-strongly interacting binary co-amorphous system carvedilol-tryptophan. Int. J. Pharm. 2022, 617, 121625 10.1016/j.ijpharm.2022.121625. 35259442

[ref29] Han J. W.; Li L. Y.; Su M. L.; Heng W. L.; Wei Y. F.; Gao Y.; Qian S. Deaggregation and Crystallization Inhibition by Small Amount of Polymer Addition for a Co-Amorphous Curcumin-Magnolol System. Pharmaceutics 2021, 13 (10), 1725 10.3390/pharmaceutics13101725. 34684018 PMC8540313

[ref30] Thakkar R.; Pillai A.; Ashour E. A.; Repka M. A. Systematic screening of pharmaceutical polymers for hot melt extrusion processing: a comprehensive review. Int. J. Pharm. 2020, 576, 118989 10.1016/j.ijpharm.2019.118989. 31931076

[ref31] Walden D. M.; Bundey Y.; Jagarapu A.; Antontsev V.; Chakravarty K.; Varshney J. Molecular Simulation and Statistical Learning Methods toward Predicting Drug-Polymer Amorphous Solid Dispersion Miscibility, Stability, and Formulation Design. Molecules 2021, 26 (1), 182 10.3390/molecules26010182. 33401494 PMC7794704

[ref32] Klamt A.; Schüürmann G. A NEW APPROACH TO DIELECTRIC SCREENING IN SOLVENTS WITH EXPLICIT EXPRESSIONS FOR THE SCREENING ENERGY AND ITS GRADIENT. J. Chem. Soc.-Perkin Trans. 2 1993, 5, 799–805. 10.1039/P29930000799.

[ref33] Klamt A. The COSMO and COSMO-RS solvation models. Wiley Interdisciplinary Reviews-Computational Molecular Science 2011, 1 (5), 699–709. 10.1002/wcms.56.

[ref34] Niederquell A.; Wyttenbach N.; Kuentz M. New prediction methods for solubility parameters based on molecular sigma profiles using pharmaceutical materials. Int. J. Pharm. 2018, 546 (1–2), 137–144. 10.1016/j.ijpharm.2018.05.033. 29772285

[ref35] Loschen C.; Klamt A. Solubility prediction, solvate and cocrystal screening as tools for rational crystal engineering. J. Pharm. Pharmacol. 2015, 67 (6), 803–811. 10.1111/jphp.12376. 25851032

[ref36] Guidetti M.; Hilfiker R.; Kuentz M.; Bauer-Brandl A.; Blatter F. Exploring the Cocrystal Landscape of Posaconazole by Combining High-Throughput Screening Experimentation with Computational Chemistry. Cryst. Growth Des 2023, 23, 842 10.1021/acs.cgd.2c01072. PMC9896487 36747574

[ref37] Chambers L. I.; Grohganz H.; Palmelund H.; Löbmann K.; Rades T.; Musa O. M.; Steed J. W. Predictive identification of co-formers in co-amorphous systems. European Journal of Pharmaceutical Sciences 2021, 157, 105636 10.1016/j.ejps.2020.105636. 33160046

[ref38] Mizoguchi R.; Waraya H.; Hirakura Y. Application of Co-Amorphous Technology for Improving the Physicochemical Properties of Amorphous Formulations. Mol. Pharmaceutics 2019, 16 (5), 2142–2152. 10.1021/acs.molpharmaceut.9b00105. 30946778

[ref39] Mathers A.; Fulem M. Drug-polymer compatibility prediction via COSMO-RS. Int. J. Pharm. 2024, 664, 124613 10.1016/j.ijpharm.2024.124613. 39179010

[ref40] Butreddy A.; Bandari S.; Repka M. A. Quality-by-design in hot melt extrusion based amorphous solid dispersions: An industrial perspective on product development. Eur. J. Pharm. Sci. 2021, 158, 105655 10.1016/j.ejps.2020.105655. 33253883 PMC7855693

[ref41] Wyttenbach N.; Niederquell A.; Ectors P.; Kuentz M. Study and Computational Modeling of Fatty Acid Effects on Drug Solubility in Lipid-Based Systems. J. Pharm. Sci. 2022, 111 (6), 1728–1738. 10.1016/j.xphs.2021.11.023. 34863971

[ref42] Abreu-Villela R.; Schönenberger M.; Caraballo I.; Kuentz M. Early stages of drug crystallization from amorphous solid dispersion via fractal analysis based on chemical imaging. Eur. J. Pharm. Biopharm. 2018, 133, 122–130. 10.1016/j.ejpb.2018.10.007. 30300718

[ref43] Li N.; Taylor L. S. Microstructure Formation for Improved Dissolution Performance of Lopinavir Amorphous Solid Dispersions. Mol. Pharmaceutics 2019, 16 (4), 1751–1765. 10.1021/acs.molpharmaceut.9b00117. 30811205

[ref44] Klamt A.; Eckert F. COSMO-RS: a novel and efficient method for the a priori prediction of thermophysical data of liquids. Fluid Phase Equilib. 2000, 172 (1), 43–72. 10.1016/S0378-3812(00)00357-5.

[ref45] Diedenhofen M.; Klamt A. COSMO-RS as a tool for property prediction of IL mixtures-A review. Fluid Phase Equilib. 2010, 294 (1–2), 31–38. 10.1016/j.fluid.2010.02.002.

[ref46] Loschen C.; Klamt A. COSMOquick: A Novel Interface for Fast sigma-Profile Composition and Its Application to COSMO-RS Solvent Screening Using Multiple Reference Solvents. Ind. Eng. Chem. Res. 2012, 51 (43), 14303–14308. 10.1021/ie3023675.

[ref47] Alin J.; Setiawan N.; Defrese M.; DiNunzio J.; Lau H.; Lupton L.; Xi H. M.; Su Y. C.; Nie H. C.; Hesse N.; et al. A novel approach for measuring room temperature enthalpy of mixing and associated solubility estimation of a drug in a polymer matrix. Polymer 2018, 135, 50–60. 10.1016/j.polymer.2017.11.056.

[ref48] Jankovic S.; Tsakiridou G.; Ditzinger F.; Koehl N. J.; Price D. J.; Ilie A. R.; Kalantzi L.; Kimpe K.; Holm R.; Nair A.; et al. Application of the solubility parameter concept to assist with oral delivery of poorly water-soluble drugs - a PEARRL review. J. Pharm. Pharmacol. 2019, 71 (4), 441–463. 10.1111/jphp.12948. 29978475

[ref49] Lin X.; Hu Y.; Liu L.; Su L. L.; Li N.; Yu J.; Tang B.; Yang Z. Y. Physical Stability of Amorphous Solid Dispersions: a Physicochemical Perspective with Thermodynamic, Kinetic and Environmental Aspects. Pharm. Res. 2018, 35 ( (6), ). DOI: 10.1007/s11095-018-2408-3. 29687226

[ref50] Greenhalgh D. J.; Williams A. C.; Timmins P.; York P. Solubility parameters as predictors of miscibility in solid dispersions. J. Pharm. Sci. 1999, 88 (11), 1182–1190. 10.1021/js9900856. 10564068

[ref51] Forster A.; Hempenstall J.; Tucker I.; Rades T. Selection of excipients for melt extrusion with two poorly water-soluble drugs by solubility parameter calculation and thermal analysis. Int. J. Pharm. 2001, 226 (1–2), 147–161. 10.1016/S0378-5173(01)00801-8. 11532578

[ref52] Yoo S. U.; Krill S. L.; Wang Z.; Telang C. Miscibility/Stability Considerations in Binary Solid Dispersion Systems Composed of Functional Excipients towards the Design of Multi-Component Amorphous Systems. J. Pharm. Sci. 2009, 98 (12), 4711–4723. 10.1002/jps.21779. 19462469

[ref53] Gómez-Carracedo A.; Alvarez-Lorenzo C.; Gómez-Amoza J. L.; Concheiro A. Chemical structure and glass transition temperature of non-ionic cellulose ethers DSC, TMDSC® -: Oscillatory rheometry study. J. Therm. Anal. Calorim. 2003, 73 (2), 587–596. 10.1023/A:1025434314396.

[ref54] Sarode A. L.; Malekar S. A.; Cote C.; Worthen D. R. Hydroxypropyl cellulose stabilizes amorphous solid dispersions of the poorly water soluble drug felodipine. Carbohydr. Polym. 2014, 112, 512–519. 10.1016/j.carbpol.2014.06.039. 25129775

[ref55] Barbooti M. M.; Alsammerrai D. A. THERMAL-DECOMPOSITION OF CITRIC-ACID. Thermochim. Acta 1986, 98, 119–126. 10.1016/0040-6031(86)87081-2.

[ref56] Shah D. S.; Takanti N.; Simpson G. J.; Taylor L. S. Orthogonal Analytical Methods to Elucidate How Low Levels of Residual Crystallinity in Posaconazole Amorphous Solid Dispersions Impact Phase Behavior during Release. Cryst. Growth Des. 2024, 24 (1), 440–454. 10.1021/acs.cgd.3c01147.

[ref57] de Waard H.; Hessels M. J. T.; Boon M.; Sjollema K. A.; Hinrichs W. L. J.; Eissens A. C.; Frijlink H. W. CLSM as Quantitative Method to Determine the Size of Drug Crystals in a Solid Dispersion. Pharm. Res. 2011, 28 (10), 2567–2574. 10.1007/s11095-011-0484-8. 21607777 PMC3170464

[ref58] Bruce C.; Fegely K. A.; Rajabi-Siahboomi A. R.; McGinity J. W. Crystal growth formation in melt extrudates. Int. J. Pharm. 2007, 341 (1–2), 162–172. 10.1016/j.ijpharm.2007.04.008. 17524578

[ref59] Chen Z.; Liu Z. S.; Qian F. Crystallization of Bifonazole and Acetaminophen within the Matrix of Semicrystalline. PEO-PPO-PEO Triblock Copolymers. Molecular Pharmaceutics 2015, 12 (2), 590–599. 10.1021/mp500661v. 25569586

[ref60] Kissi E. O.; Khorami K.; Rades T. Determination of Stable Co-Amorphous Drug-Drug Ratios from the Eutectic Behavior of Crystalline Physical Mixtures. Pharmaceutics 2019, 11 (12), 628 10.3390/pharmaceutics11120628. 31771255 PMC6956160

[ref61] Luebbert C.; Wessner M.; Sadowski G. Mutual Impact of Phase Separation/Crystallization and Water Sorption in Amorphous Solid Dispersions. Mol. Pharmaceutics 2018, 15 (2), 669–678. 10.1021/acs.molpharmaceut.7b01076. 29309155

[ref62] Edueng K.; Bergström C. A. S.; Gråsjö J.; Mahlin D. Long-Term Physical (In)Stability of Spray-Dried Amorphous Drugs: Relationship with Glass-Forming Ability and Physicochemical Properties. Pharmaceutics 2019, 11 (9), 425 10.3390/pharmaceutics11090425. 31438566 PMC6781026

[ref63] Zografi G.; Newman A. Interrelationships Between Structure and the Properties of Amorphous Solids of Pharmaceutical Interest. J. Pharm. Sci. 2017, 106 (1), 5–27. 10.1016/j.xphs.2016.05.001. 27372552

[ref64] Bhattacharya S.; Suryanarayanan R. Local Mobility in Amorphous Pharmaceuticals-Characterization and Implications on Stability. J. Pharm. Sci. 2009, 98 (9), 2935–2953. 10.1002/jps.21728. 19499564

